# The impact of lifestyle counselling on weight management and quality of life among working-age females

**DOI:** 10.1080/02813432.2021.1958510

**Published:** 2021-09-03

**Authors:** Jenni Virtanen, Markus Penttinen, Hannu Kautiainen, Päivi Korhonen

**Affiliations:** a Department of General Practice, Turku University and Turku University Hospital, Turku, Finland; b Perusturvakuntayhtyma Akseli, Masku, Finland; c Terveystalo, Turku, Finland; d Folkhälsan Research Center, Helsinki, Finland; e Unit of Primary Health Care, Kuopio University Hospital, Kuopio, Finland

**Keywords:** Lifestyle counselling, weight management, weight loss, primary health care, overweight, obesity, quality of life

## Abstract

**Objective:**

Overweight and obesity are increasing globally. General practitioners (GP’s) are at the first point of contact for medical support and consequently have a major role in resolving this overwhelming problem. The aim of this study was to assess the effectiveness of a brief lifestyle counselling on weight management and on the participants’ quality of life (QoL).

**Design:**

A cohort study with a one-year follow-up.

**Setting:**

Occupational health care, city of Pori in southwestern Finland.

**Participants:**

Female municipal employees (*n* = 625) with a mean age of 48 (SD 9) years.

**Intervention:**

A nurse and a physiotherapist gave lifestyle counselling to all the participants; however, only the overweight/obese subjects were recommended to lose at least 5% of their initial weight.

**Main outcome measure:**

Success in weight management and quality of life.

**Results:**

At the follow-up visit, 10.4% (95% CI: 7.5–14.0) of the overweight/obese subjects had lost at least 5% of their weight, but 10.0% (95% CI: 6.7–14.3) of the normal-weight participants had become overweight. The mean weight change was +0.1 kg (95% CI: −0.3–0.5) in the overweight/obese group and +0.5 kg (95% CI: 0.2–0.8) in the normal weight group. The change in QoL was inversely correlated with relative weight change in overweight/obese subjects, albeit the effect size was small.

**Conclusion:**

Weight management counselling should also be directed to individuals with a normal weight. Even with brief lifestyle counselling it may be possible to stabilize weight gain. Successful weight loss may improve the QoL of overweight/obese individuals.

**KEY POINTS**
- Primary health care has to deal with the increasing problem of overweight and obesity.
- Brief lifestyle counselling performed by a nurse and a physiotherapist seems to be quite effective in weight stabilization, considering the effort needed.
- People with normal weight tend to gain weight and weight management counselling should also be directed to them. Successful weight management may improve the quality of life of overweight/obese people.

## Introduction

Globally, overweight and obesity are huge and increasing problems. In the OECD countries, 20% of adults were obese and more than 50% were overweight in the year 2015 [1]. In Finland, 72% of men and 63% of women aged 30 years or over are overweight or obese [2]. Furthermore, the rates of overweight/obesity are expected to rise in the future [1]. This is alarming since a high body-mass index (BMI) is a risk factor for many chronic diseases such as cardiovascular diseases, type 2 diabetes, cancer, and musculoskeletal disorders [3].

In addition to these health-related problems, overweight/obesity also has a negative impact on people’s quality of life (QoL) [4]. There are numerous studies and reviews – even reviews of reviews – about overweight/obesity, weight management, and its impact on health-related QoL (HRQoL) [4]. The current knowledge is that improvement in HRQoL has been demonstrated following bariatric surgery but not with non-surgical weight loss interventions [4].

Successful weight loss interventions often require resources that are seldom feasible in primary health care [5]. However, general practitioners (GPs) are at the first point of medical contact and therefore taking responsibility for the consequences of overweight/obesity. There is a need for a rapid, inexpensive, and effective intervention for the treatment of overweight/obesity.

Finland has a special occupational health care system, which is intended to prevent work-related illnesses and accidents and to promote employees' work capacity and functioning. These services are free for employees and paid for by the employer. Occupational health care offers a suitable setting for GPs and nurses to provide lifestyle counselling including advice on weight management.

The aim of the present study was to assess the effectiveness of lifestyle counselling on weight management in a feasible setting in primary health care. We were also interested in the impact of weight change on QoL. To obtain a more comprehensive understanding of the subjects’ well-being than merely using an HRQoL scale, we employed the generic EUROHIS-QoL 8-item instrument [6]. We hypothesized that lifestyle counselling could be effective in weight management and result in a positive change in the subjects’ QoL.

## Material and methods

### Participants

The study is part of the PORTAAT-study (PORi To Aid Against Threats), which is a longitudinal cohort study conducted among municipal employees in the city of Pori (83,497 inhabitants in 2014) in southwestern Finland in 2014–2015. The study population was derived from 10 work units selected by the head of the welfare unit in Pori. Invitations to participate in the study and information letters were sent to employees (*n* = 2570) as an email attachment by the managers of the work units. In total, 836 employees (104 men, 732 women) consented to participate in the study, the response rate being 33%. Study participants represented an active workforce and a variety of professions: librarians, museum employees, groundkeepers, computer workers, social workers, nurses, physicians, administrative officials, and general office staff. There were no exclusion criteria.

### Enrollment visit

In 2014, the employees were invited to an enrollment visit performed by a trained study nurse and given self-administrated questionnaires to be completed at home.

### Questionnaires

Quality of life (QoL) was assessed with a generic EUROHIS-QoL 8-item index, which is a shortened version of the WHOQoL-Bref–scale [7]. The EUROHIS-QoL instrument has been validated in several European countries and it can be used to measure QoL in epidemiologic surveys [8]. Every question was scored from 1 to 5 (1 for very poor and 5 for very good). All scores were then added together and divided by 8 (the sum of the questions) to obtain the EUROHIS-QoL mean score. The eight questions of EUROHIS-QoL instrument are:

- How would you rate your quality of life?
- How satisfied are you with your health?
- How satisfied are you with your ability to perform your daily living activities?
- How satisfied are you with yourself?
- How satisfied are you with your personal relationships?
- How satisfied are you with the conditions of your living place?
- Do you have enough money to meet your needs?
- Do you have enough energy for everyday life?

Regular physical activity (PA) was assessed with a self-administrated questionnaire with the frequency and duration of leisure time and commuting activities in a typical week. Fulfilling the national recommendations for physical activity was defined as engaging in ≥150 min per week of moderately intense activities (i.e. brisk walking) or ≥75 min per week of vigorously intense (i.e. running) activities [9].

Information on diet was collected with a food-frequency questionnaire including consumption of fruits, vegetables, whole grains, dairy products, fish, and meat.

Smoking status was assessed by a questionnaire. Non-smoking was defined as having never smoked or having quit smoking >12 months ago. Alcohol consumption was assessed using the 3-item Alcohol Use Disorders Identification Test (AUDIT-C) with a cut-off of 5 points for harmful alcohol use in women and 6 points in men [10,11]. Information about the years of education, marital status (cohabiting or not), financial satisfaction (with the question “I have to spare in my expenditures”, yes or no), and sleep quality (good or poor) were collected from self-administrated questionnaires.

### Baseline examination and lifestyle counselling

#### Baseline examination

Height and weight were measured with subjects in a standing position without shoes and outer garments. Weight was measured to the nearest 0.1 kg with calibrated scales and height to the nearest 0.5 cm with a wall-mounted stadiometer. BMI was calculated as weight (kg) divided by the square of height (m^2^). Overweight was defined as a BMI of 25.0–29.9 kg/m^2^ and obesity ≥30.0 kg/m^2^ [12]. Blood pressure (BP) was measured with an automatically validated blood pressure monitor with subjects in a sitting posture after resting for at least 5 min. Two readings taken at intervals of at least 2 min were measured, and the mean of these readings was used in the analysis. Every subject had his/her test results written down in a notebook along with target values.

Psychosocial risk factors were assessed by core questions suggested by the European 2012 Guidelines on CVD Prevention in Clinical Practice [13]:

- **Work and family stress**. Do you have enough control over how to meet the demands at work? Is your reward appropriate for your effort? Do you have serious problems with your spouse?
- **Depression**. Do you feel down, depressed and hopeless? Have you lost interest and pleasure in life?
- **Anxiety**. Do you frequently feel nervous, anxious, or on edge? Are you frequently unable to stop or control worrying?
- **Hostility**. Do you frequently feel angry over little things? Do you often feel annoyed about habits other people have?
- **Social isolation**. Are you living alone? Do you lack a close confidant?

If a subject answered “yes” to one or more of these questions it indicated a likely psychosocial risk factor.

Previously diagnosed diseases and regular medication were obtained from the questionnaires and medical records. Laboratory tests were determined from blood samples, which were obtained after at least 8 h of fasting. Total cholesterol, high-density lipoprotein cholesterol (HDL-C), and triglycerides were measured enzymatically (Architect c4000/c8000). Low-density lipoprotein cholesterol (LDL-C) was calculated by Friedewald’s formula. Glucose tolerance was measured with glycated hemoglobin (HbA1c) which was analyzed using a High-Performance Liquid Chromatography -method (Tosoh HLC-723G7).

#### Lifestyle counselling

Based on the baseline examination, food frequency questionnaire, and reported lifestyle habits, the study subjects received diet and lifestyle counselling from the study nurse. The main recommendations were to avoid fatty ingredients, prefer whole grain products, fruits and vegetables, and use fish and poultry rather than red meat. For overweight/obese subjects (BMI ≥25.0 kg/m^2^) the main goal was a weight reduction of at least 5%.

In addition, a trained physiotherapist had a discussion with every participant and gave individual advice as regards performing physical activity (PA). If the participant’s PA was not at the recommended level, they were advised to perform moderately intense PA at least 30 min per day.

### Control visit in 2015

After a 1-year follow-up time, the subjects were invited to a control appointment with the study nurse. The study procedures and the questionnaire surveys, with the exception of the food frequency questionnaire, were repeated at the baseline visit in 2014.

**Figure 1.**
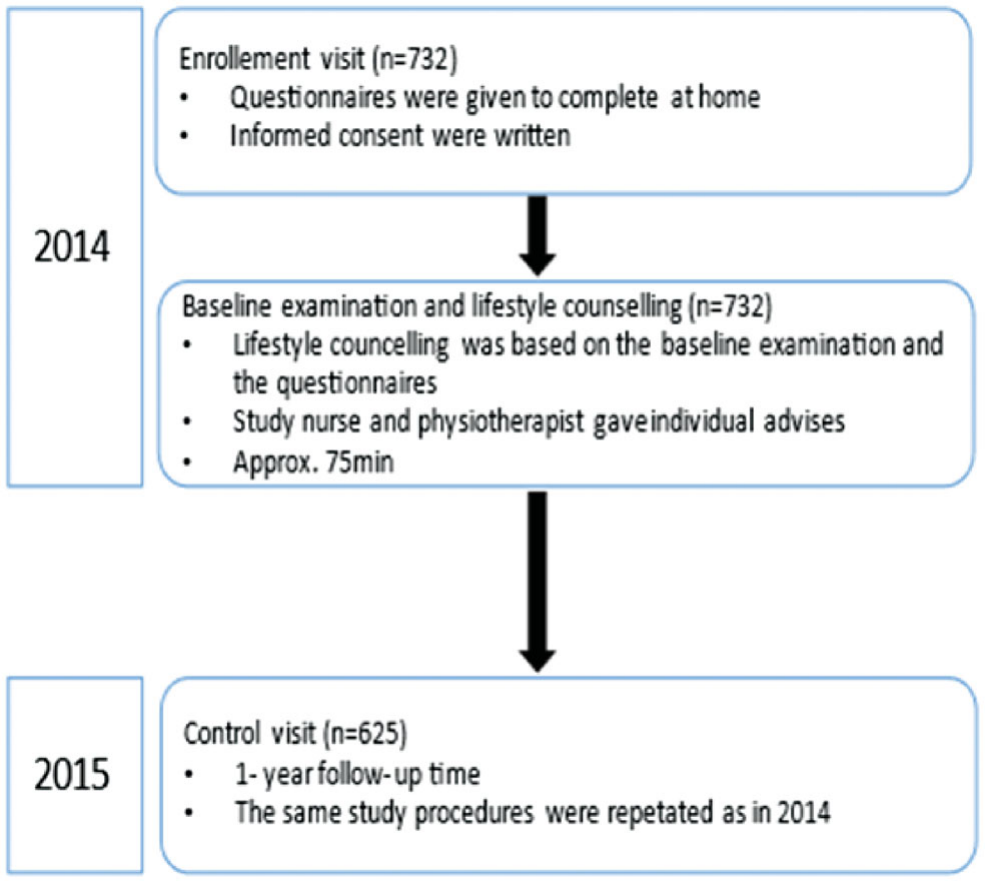
Demonstrating flow of the study.

For the present analyses, we reported data from 625 female participants who completed the follow-up in 2015. Because there were so few male participants (*n* = 103), we restricted the analyses to only the women. The study protocol is presented in Figure 1.

#### Informed consent and ethical approval

The study protocol and consent forms were reviewed and approved by the Ethics Committee of the Hospital District of Southwest Finland. All participants provided written informed consent for the project and subsequent medical research.

### Statistical analysis

Data are presented as means with standard deviation (SD) and as counts with percentages. The relationships of dichotomized weight change (lose weight or stabilized/gained weight) and EUROHIS-8 change categories (QoL reduced or didn’t change/QoL improved) with background characteristics were analyzed using generalized linear models. The main and interactive effects of weight change and QoL change were analyzed by entering dichotomized variables and their interaction as independent variables into the models. In the case of violation of the assumptions (e.g. non-normality), a bootstrap-type test was used. The relationship between relative weight change and change of EUROHIS-8 was modeled using linear regression analysis. Cohen’s effect-size statistics (*f^2^*) was used as an indicator of the strength of relative weight change effects on changes in the EUROHIS-8. Cohen’s standard for effect size values above 0.02, 0.15, and 0.35 represent small, moderate, and large effect sizes, respectively. The normality of the variables was evaluated graphically and using the Shapiro–Wilk *W* test. A Stata 16.0 (StataCorp LP, College Station, TX, USA) was used for the analysis.

## Results

**Figure 2.**
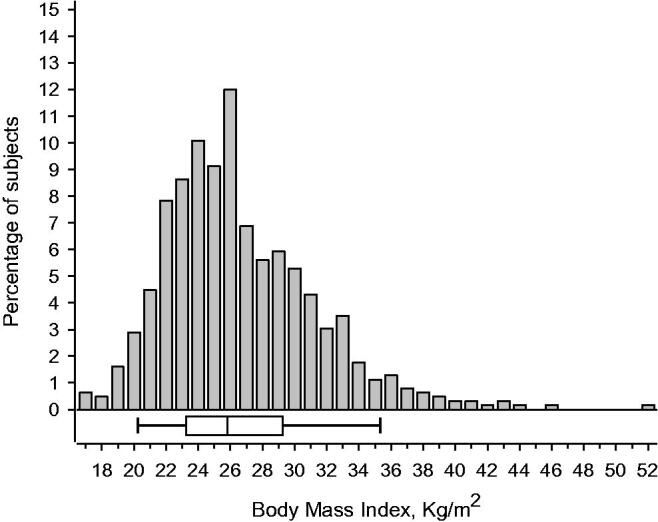
Distribution of the Body Mass Index in the study population. Box-and-whiskers plot shows median and IQR (interquartile range), and whiskers indicate 5th and 95th percentile.

Lifestyle counselling was given to 625 female municipal employees (mean age 48 ± 9 years), of whom 366 (58.6%) were overweight/obese; these participants were also recommended to reduce their weight by at least 5%. A histogram of the distribution of BMI in the study population is presented in Figure 2.

Characteristics of the subjects according to categories of weight change and change in EUROHIS-8 are presented in Table 1. The only statistical difference detected was that women who managed to reduce or stabilize their weight were older than those who gained weight during the follow-up.

**Table 1.** Characteristics of the subjects according to categories of weight change and change in the quality of life (EUROHIS-8).

	Weight change						
	Reduced or stabilized		Gained weight		*P* value		
Demographic factors	QoL reduced or no change*N* = 114	QoL improved*N* = 163	QoL reduced or no change*N* = 142	QoL improved*N* = 206	Weight change	QoL change	Interaction
Age, years, mean (SD)	49 (9)	48 (9)	47 (11)	47 (10)	0.042	0.28	0.69
Education years, mean (SD)	13.8 (2.1)	13.7 (2.1)	13.8 (2.2)	13.7 (2.2)	0.91	0.74	0.96
Cohabiting, *n* (%)	91 (80)	128 (79)	108 (76)	166 (81)	0.82	0.64	0.39
Lifestyle factors							
Current smokers, *n* (%)	14 (12)	21 (13)	10 (7)	20 (10)	0.088	0.46	0.59
AUDIT-C, mean (SD)	3.0 (1.7)	2.9 (1.7)	3.2 (1.9)	3.1 (2.1)	0.099	0.44	0.90
Physical activity at goal, *n* (%)	48 (42)	65 (40)	61 (43)	82 (40)	0.92	0.50	0.91
Financial satisfaction, *n* (%)	72 (63)	99 (61)	90 (63)	131 (64)	0.70	0.78	0.74
Good quality of sleep, *n* (%)	86 (75)	112 (69)	96 (68)	141 (68)	0.27	0.41	0.30
Laboratory measures, mean (SD)							
Total cholesterol, mmol/l	5.28 (0.85)	5.32 (0.90)	5.27 (0.91)	5.22 (0.99)	0.44	0.95	0.52
LDL cholesterol, mmol/l	3.05 (0.73)	3.07 (0.78)	3.02 (0.74)	2.93 (0.84)	0.19	0.60	0.42
HDL cholesterol, mmol/l	1.76 (0.46)	1.75 (0.46)	1.77 (0.45)	1.80 (0.43)	0.40	0.83	0.54
Triglycerides, mmol/l	1.06 (0.56)	1.13 (0.67)	1.10 (0.46)	1.05 (0.52)	0.61	0.73	0.17
HbA1c, mmol/mol	5.48 (0.50)	5.47 (0.46)	5.40 (0.31)	5.45 (0.48)	0.21	0.58	0.46
Clinical measures, mean (SD)							
Height, cm	165 (7)	165 (6)	164 (6)	165 (6)	0.66	0.37	0.33
Weight, kg	71.7 (12.2)	73.9 (14.6)	72.8 (15.3)	72.0 (13.5)	0.75	0.55	0.18
Body mass index, kg/m^2^	26.2 (4.1)	27.0 (4.8)	26.8 (5.1)	26.3 (4.8)	0.88	0.74	0.11
Systolic blood pressure, mmHg	133 (17)	132 (17)	130 (16)	130 (18)	0.12	0.86	0.56
Diastolic blood pressure, mmHg	85 (10)	87 (11)	85 (11)	85 (10)	0.36	0.63	0.26
Number of chronic diseases	0.9 (1.1)	1.0 (1.1)	1.0 (1.1)	0.9 (1.1)	0.99	0.75	0.62
Medication, *n* (%)							
Antidiabetic	5 (4)	7 (4)	5 (4)	9 (4)	0.80	0.81	0.77
Antihypertensive	17 (15)	35 (21)	30 (21)	45 (22)	0.29	0.25	0.34
Statins	6 (5)	6 (4)	7 (5)	10 (5)	0.78	0.62	0.65
Psychosocial risk factors, *n* (%)							
Depression	15 (13)	33 (20)	26 (18)	41 (20)	0.40	0.16	0.35
Stress	30 (26)	47 (29)	50 (35)	57 (28)	0.32	0.53	0.19
Social isolation	16 (14)	26 (16)	30 (21)	35 (17)	0.20	0.79	0.34
Anxiety	32 (28)	46 (28)	46 (32)	69 (33)	0.20	0.87	0.91
Hostility	19 (17)	33 (20)	35 (25)	46 (22)	0.13	0.79	0.37

QoL: quality of life; AUDIT-C: Alcohol Use Disorders Identification Test; LDL: low-density lipoprotein; HDL: high-density lipoprotein; HbA1c: glycated hemoglobin.

At the one-year follow-up visit, 38/366 [10.4% (95% CI: 7.5–14.0)] of the overweight/obese subjects had lost at least 5% of their initial weight. Of the normal-weight subjects at baseline, 26/259 [10.0% (95% CI: 6.7–14.3)] had a BMI of ≥25.0 kg/m^2^ at the follow-up visit.

The mean weight change (follow-up to baseline) was + 0.1 kg (95% CI: −0.3–0.5, *p* = 0.66) in the overweight/obese group, and + 0.5 kg (95% CI: 0.2–0.8, *p* < 0.001) in the normal weight group.

The mean change in the EUROHIS-8 score was 0.12 (95% CI: 0.08–0.16, *p* < 0.001) among the overweight/obese subjects and 0.12 (95% CI: 0.08–0.16, *p* < 0.001) among the normal-weight subjects.

**Figure 3.**
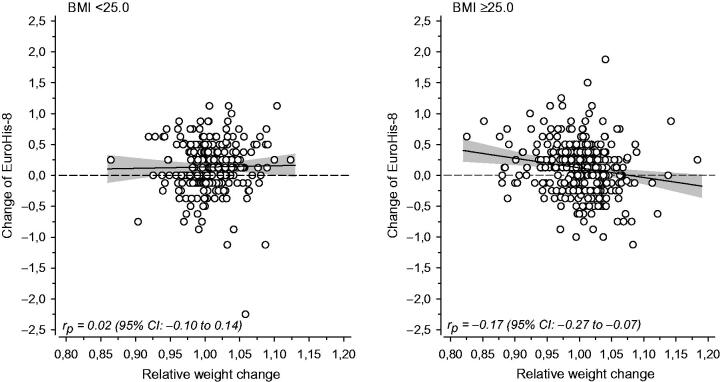
The relationship between the change of EUROHIS-8 score and the relative weight change among normal weight (BMI <25 kg/m^2^) and overweight/obese subjects (BMI ≥25 kg/m^2^) at baseline. The line shows the estimated linear regression with 95% confidence intervals.

Figure 3 shows the relationship between the change of EUROHIS-8 score and the relative weight change among normal weight and overweight/obese subjects at baseline. The change in QoL was inversely correlated with relative weight change only in overweight/obese subjects (*p* = 0.025), the partial correlation coefficient being −0.17 (95% CI: −0.27 to −0.07). The effect size measured by Cohen’s *f*^2^ was small: 0.03 (95% CI: 0.01–0.08) for overweight/obese subjects and 0.00 (95% CI: 0–0.02) for normal-weight subjects.

## Discussion

The main finding of this study is that with brief, two-stage lifestyle counselling sessions given by a nurse and a physiotherapist, one in ten of the overweight/obese subjects succeeded in losing at least 5% of their weight, as recommended. The mean weight gain in the overweight/obese group was 0.1 kg per year, indicating that most of the participants were able to stabilize their weight. However, one in ten of the normal weight subjects at baseline also gained weight so that they were overweight at the one-year follow-up visit. Interestingly, QoL increased among the overweight/obese subjects who managed to lose weight, but among the normal weight group, QoL did not change according to their relative weight change.

As demonstrated in Table 1, the only statistically significant variable in weight change was age although this appears not be a clinically meaningful difference. Thus, we are not able to demonstrate any single factor that would be beneficial for weight loss or any subgroup of the subjects who could benefit from the intervention.

Nowadays, adults tend to gain weight at ∼0.4–1 kg per year [14–16], overweight and obese people tend to gain even more than those with a normal BMI [16]. In our study, participants in the normal weight group gained 0.5 kg per year on average, even though they had the same lifestyle counselling as the overweight/obese participants. The only difference in lifestyle counselling between the groups was that the overweight/obese subjects were also recommended to lose weight. In conclusion, the normal weight subjects would have probably benefitted from more precise and strict instructions as regards retaining their current weight and avoiding weight gain in the future. Indeed, the World Health Organization (WHO) has suggested that weight management should concentrate on prevention, and not only weight loss [17].

To our knowledge, generic QoL instruments are rarely used to assess overall QoL in weight management interventions. We decided to use a generic instrument instead of a condition-specific health-related scale considering the quite healthy study population. In addition, a generic QoL instrument demonstrates the individual’s overall life satisfaction, not only health-related satisfaction. It’s well known that people with low socioeconomic status have a higher risk for overweight/obesity and the EUROHIS-QoL instrument has also questions about living place and financial satisfaction [18].

We found only one study using items from the EUROHIS-QoL instrument. In this study, conducted among the general Polish population, weight loss was associated with a better QoL [19]. In line with that study, our results suggest that there was a small inverse correlation between weight change and change in QoL among the overweight/obese participants (Figure 3). What is more surprising is that there was no relationship between relative weight change and change in QoL in the normal weight group. If gaining weight does not affect QoL when the person is still at a normal weight or slightly overweight, then they may not be aware of any need for a change in their lifestyle. This could be one reason why people with normal weight tend to gain weight. If a small weight gain does not affect people’s QoL, they may not notice it.

The main outcome measure, which only one in ten overweight/obese participants succeeded in achieving was a 5% weight reduction, which may not be considered the desired result for a weight management intervention. However, considering the large number of subjects who managed to stabilize their weight and the rather small amount of effort needed by public health care, the achieved result can be regarded as fairly good. In a Swedish study by Waller et al. using a quite similar lifestyle counselling setting, 47% of the subjects aged 18–79 were able to improve their BMI in the one-year follow-up time [20]. This is comparable to results observed in our study (42% BMI reduction among 18–65 year-old subjects). These results provide encouragement to continue lifestyle counselling in primary health care, especially for the prevention of weight gain.

## Strengths and limitations

The strengths of the study are that there was quite a large study population and a long follow-up time, so the weight change results can be regarded as permanent. The clinical measures were performed by a trained study nurse as well the lifestyle counselling. No GPs contributed to the study, so the lifestyle counselling setting can be applied in primary health care with limited resources. There are also limitations in this study. Firstly, the study population only consisted of females, so the results cannot be generalized to males. Secondly, the response rate was only 33%. It is possible that some employees ignored the invitation letters sent *via* e-mail. E-mail surveys generally have ∼20% lower response rates than mail surveys [21]. It is also possible that there is selection bias regarding our study population, individuals with poor lifestyle habits may not have been willing to participate. Thirdly, the correlation between weight change and QoL was rather small and there may be other factors contributing to this result besides weight change. In addition, the results pertaining to the weight change for the overweight and the normal-weight group may be the result of regression towards the mean.

## Conclusion

In conclusion, if weight management programs are only directed towards overweight/obese individuals, the rates of overweight/obesity will keep rising since the study shows that a large number of normal-weight people can become overweight during just a one-year period. Thus, overweight prevention should be the main target in population-level strategies. In primary health care, nurse-led lifestyle counselling with a recommendation to lose weight may also improve QoL among overweight/obese individuals who succeed in weight management. On the other hand, weight gain may reduce the quality of life among overweight/obese individuals.
